# Characterization of aEEG During Sleep and Wakefulness in Healthy Children

**DOI:** 10.3389/fped.2021.773188

**Published:** 2022-01-21

**Authors:** Verena T. Löffelhardt, Adela Della Marina, Sandra Greve, Hanna Müller, Ursula Felderhoff-Müser, Christian Dohna-Schwake, Nora Bruns

**Affiliations:** ^1^Department of Pediatrics I, Neonatology, Pediatric Intensive Care Medicine, and Pediatric Neurology, University Hospital Essen, University of Duisburg-Essen, Essen, Germany; ^2^TNBS, Centre for Translational Neuro- and Behavioral Sciences, University Hospital Essen, University of Duisburg-Essen, Essen, Germany; ^3^Department of Pediatrics, Neonatology and Pediatric Intensive Care, University of Marburg, Marburg, Germany

**Keywords:** amplitude-integrated EEG (aEEG), sleep states, children, sleep, antiepileptic drug (AED), wakefulness

## Abstract

**Introduction:**

Interpretation of amplitude-integrated EEG (aEEG) is hindered by lacking knowledge on physiological background patterns in children. The aim of this study was to find out whether aEEG differs between wakefulness and sleep in children.

**Methods:**

Forty continuous full-channel EEGs (cEEG) recorded during the afternoon and overnight in patients <18 years of age without pathologies or only solitary interictal epileptiform discharges were converted into aEEGs. Upper and lower amplitudes of the C3–C4, P3–P4, C3–P3, C4–P4, and Fp1–Fp2 channels were measured during wakefulness and sleep by two investigators and bandwidths (BW) calculated. Sleep states were assessed according to the American Academy of Sleep Medicine. Median and interquartile ranges (IQR) were calculated to compare the values of amplitudes and bandwidth between wakefulness and sleep.

**Results:**

Median age was 9.9 years (IQR 6.1–14.7). All patients displayed continuous background patterns. Amplitudes and BW differed between wakefulness and sleep with median amplitude values of the C3–C4 channel 35 μV (IQR: 27–49) for the upper and 13 μV (10–19) for the lower amplitude. The BW was 29 μV (21–34). During sleep, episodes with high amplitudes [upper: 99 μV (71–125), lower: 35 μV (25–44), BW 63 μV (44–81)] corresponded to sleep states N2–N3. High amplitude-sections were interrupted by low amplitude-sections, which became the longer toward the morning [upper amplitude: 39 μV (30–51), lower: 16 μV (11–20), BW 23 μV (19–31)]. Low amplitude-sections were associated with sleep states REM, N1, and N2. With increasing age, amplitudes and bandwidths declined.

**Conclusion:**

aEEGs in non-critically ill children displayed a wide range of amplitudes and bandwidths. Amplitudes were low during wakefulness and light sleep and high during deep sleep. Interpretation of pediatric aEEG background patterns must take into account the state of wakefulness in in clinical practice and research.

## Introduction

The use of amplitude-integrated electroencephalography (aEEG), a simplified time-compressed EEG, to continuously monitor cerebral function is increasing in pediatric intensive care (1, 2). Growing evidence in favor of continuous electroencephalography (cEEG) monitoring, limited access to full channel cEEG, and broad availability of aEEG devices in neonatal intensive care units promote this development (2–5). While aEEG monitoring is well-established in preterm infants and neonates, recent evidence suggests that aEEG helps to identify seizures in older children on the pediatric intensive care unit (PICU) (6–10). Furthermore, analysis of the background pattern may aid in predicting outcomes pediatric after cardiac arrest (11).

Studies on normal background patterns and amplitude values in newborns showed that variations of the upper and lower amplitudes concur with changes in wakefulness (12–15). These variations are referred to as sleep-wake cycling and have to be taken into account when interpreting neonatal aEEGs. The presence or absence of sleep-wake cycling is associated with outcomes in several diseases in neonates and young infants up to 3 months of age (16–18). In older infants and children, sleep patterns have been studied by 24 h EEG and polysomnography. Amplitude height is used among other information to determine the sleep state from raw EEG, with lower amplitudes during light sleep or rapid-eye-movement (REM) sleep and higher amplitudes during deep sleep. Whether these amplitude variations in the raw EEG translate into a sleep-wake cycling pattern that can be recognized by aEEG has not yet been investigated.

For interpretation of aEEG background patterns, lacking reference values for infants and children constitute a serious challenge for clinicians (1, 2). A continuous background pattern with lower border values above 5–7 μV is generally considered normal (6, 8, 19). However, these values derive from a classification proposed for term born and preterm infants by Hellström-Westas, which remains unvalidated in older infants or children (13). For this reason, aEEG interpretation in the pediatric intensive care setting mainly focuses on the detection of seizures and severe abnormalities (2, 6–8, 10).

Defining normal values for children to facilitate aEEG interpretation and promote the identification of abnormal electrocortical activity is one of the major steps ahead in research on pediatric aEEG. However, without knowledge on physiological intra-individual amplitude variations, normal values cannot be defined. The aim of this study was to explore whether aEEG background activity differs between wakefulness and sleep in non-critically ill children. For this purpose, we analyzed pediatric long-term EEGs from our neurophysiology department after converting them into aEEGs.

## Methods

### Inclusion Criteria

The indication for continuous full-channel EEGs (cEEG) recording from the afternoon until the next morning was set by the attending pediatric neurologist. For this study, findings of cEEGs conducted between 2017 and 2020 in patients <18 years of age in the pediatric neurology department of the University Hospital Essen were screened by retrospective chart review. cEEGs that had been rated as containing no pathology or only solitary interictal epileptiform discharges (IEDs) by the attending pediatric neurologist were eligible for conversion to aEEG and subsequent analysis. Pathological recordings with documented repeated IEDs or impaired sleep architecture were not eligible. Clinical information was extracted from patient charts.

### Continuous EEG Recording

cEEG were recorded using Polaris EEG software (Nihon Kohden, Japan). Upon hospital admission, full-channel EEG (cEEG) was applied in the early afternoon by the attending EEG nurse according to the international 10–20 system. After skin preparation with OneStep EEG Gel Abrasiv plus^®^ and application of Elefix conductive EEG paste (Nihon Kohden), the electrodes were placed. Impedance check was performed, and skin preparation repeated until impedances below <50 kΩ were achieved for all electrodes, before carrying out the recording until the next morning. The parents or patients themselves recorded all activities during the recording, e.g., medical exams, meals, going to bed, and clinical or suspected seizures. No sedations or invasive procedures were performed during the recording. Medical exams conducted were ultrasound, electrocardiograms, or other non-invasive diagnostics.

### aEEG Analysis

The entire cEEG tracings were converted into aEEGs using an extension of the Polaris EEG software (Polaris Trend Software QP-160AK, Nihon Kohden, Tokyo, Japan). To convert the EEG signal into an aEEG, the raw EEG signal was amplified, passed through a band-pass filter, transformed logarithmically, and finally rectified as described by Maynard et al. (20). We converted the channels C3–C4, P3–P4, C3–P3, C4–P4, and Fp1–Fp2 of the 10–20 system because these channels are used in pediatric intensive care when applying single- or two-channel aEEGs.

**Figure 1:**
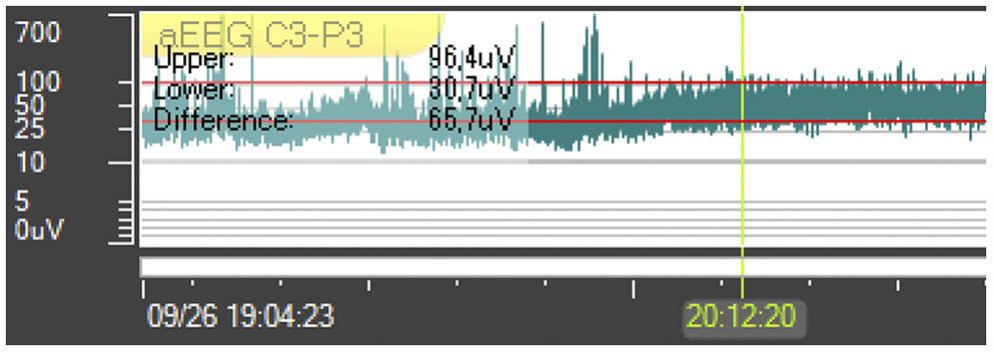
Measurement of the amplitudes. The vertical green line can be manually moved to the beginning of the measurement section. To measure the amplitudes, the horizontal red lines are manually aligned with the upper and lower borders. The values are displayed in the box in the upper left corner.

VL and NB measured the height of the upper and lower borders of amplitude (μV) of all channels manually within the Trend Program. The tool for amplitude measurement functions like a ruler that is positioned parallel to the upper and lower border manually, displaying the numerical value of the upper and lower amplitudes and the bandwidth in μV (Figure 1). A cursor was set to mark the beginning of each analyzed section (Figure 1).

**Figure 2:**
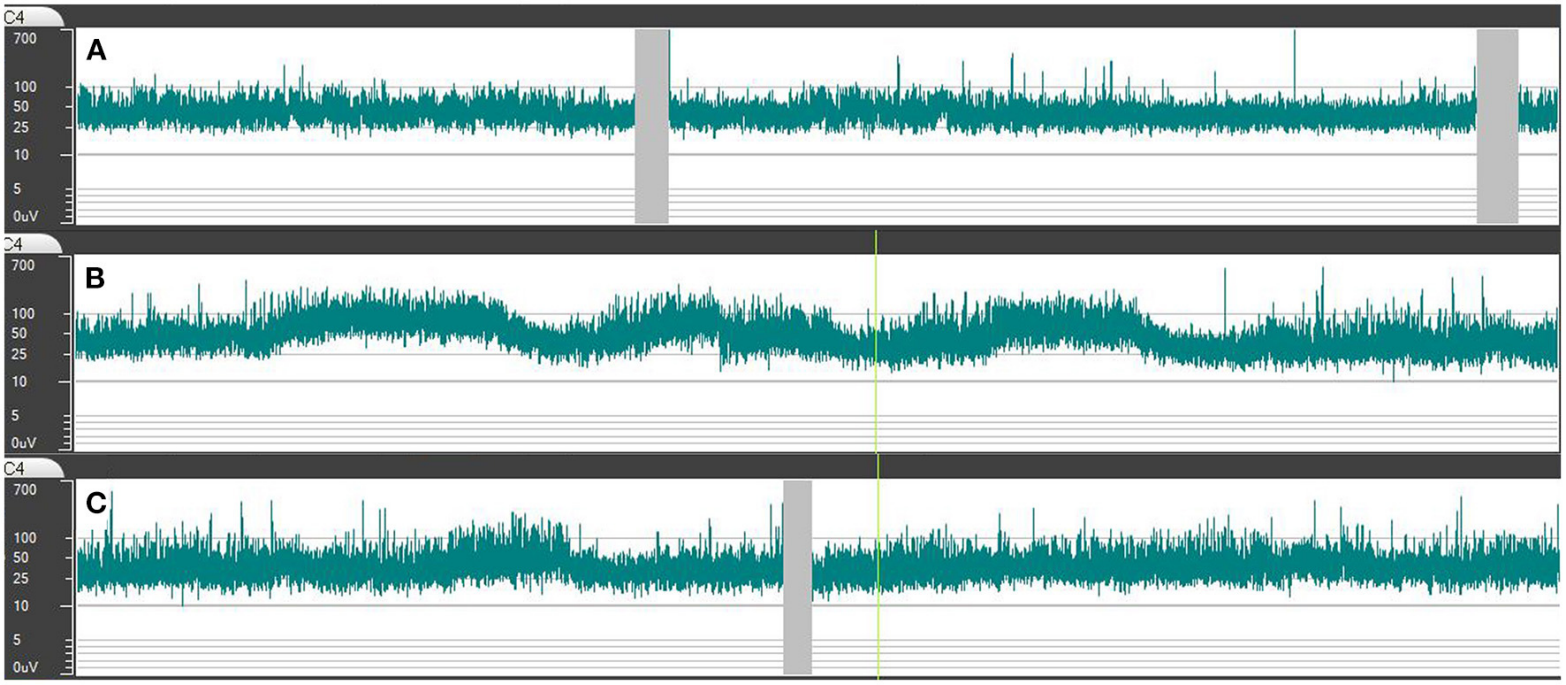
Representative course of amplitudes and bandwidth during recordings of the C3–C4 channel. Simultaneous changes were observed in all analyzed channels. **(A)** During wakefulness, the aEEG band shows a continuous background pattern with rather low amplitudes and bandwidth. **(B)** In the first half of the night, a rise in the upper and lower amplitude occurs. The increase in bandwidth is less obvious due to the logarithmic scale of the vertical axis. **(C)** Toward the morning, the amplitudes and bandwidth lower again.

To develop an understanding how the aEEG band varies throughout the day, several tracings were visually reviewed before systematic assessment. This preliminary visual assessment showed, that during wakefulness (as documented by the parents or patients in the chart) the aEEG band showed little or no variation of the upper lower amplitudes. After going to bed, the aEEG band rose to higher amplitude values, displaying intermittent dips with lower amplitudes throughout the night. Toward the morning, aEEG bands lowered again in all tracings (Figure 2). Because of this finding, we assessed three different sections within each tracing:

- Wakefulness: 1 h of artifact-free recording in the afternoon was measured.
- Sleep: For measurement of sleep amplitudes, the period between going to bed (as documented in the chart) and five a.m. was eligible. At that time, noises begin to rise in our hospital and sleep is disturbed.
  ° High amplitude-section: Amplitudes of the first 30 min section with high aEEG band were measured.
  ° Low-amplitude section: For low amplitude measurement, the section with the lowest amplitudes during sleep was assessed. If this section was shorter than 30 min, then the nadir of the dip was measured.

The beginning of each measured section was labeled in the tracing, and the measured values were displayed at the side. An image of the measured and labeled tracing was then saved as a PDF file for later assessment of interrater agreement and potential measurement errors. The measured values were transferred manually into an excel spreadsheet.

### cEEG Review

All cEEGs that fulfilled the inclusion criteria were reviewed again by a board-certified pediatric neurologist (ADM) blinded to aEEG findings (certificates in EEG and epileptology by the German Society for Epileptology). If interictal epileptiform discharges or other pathologies were detected during any interval of interest, the recording was retrospectively excluded.

The time intervals identified from aEEGs during sleep (30 min) were reviewed and the sleep state was determined according to the scoring system by the American Academy of Sleep Medicine (21). In case the sleep state changed during the reviewed interval, both sleep states were documented. As our recordings did not contain electro-oculography, REM sleep, and N1 could not always be clearly differentiated. This was documented as “N1 or REM.”

### Data Analysis

Continuous variables are presented as median and interquartile range (IQR). The mean ± standard deviation (SD) is given where information on scattering of the values is of interest. The mean and 95% confidence intervals (CI) are given in the sensitivity analysis (see below) to enable comparison between groups. Ninety five percent prediction limits (the range in which 95% of future measurements would be expected) were calculated based on the mean and standard deviation. For variables with non-normally distributed data, logarithmic transformation was performed before calculation of confidence intervals and prediction limits (upper and lower amplitudes and bandwidths of Fp1–Fp2).

For discrete variables, absolute and relative frequencies are given.

#### Calculation of Amplitudes and Bandwidth

To determine the amplitude of the upper and lower amplitudes of each aEEG channel, we calculated the mean of the measurements by VL and NB. The bandwidth was calculated as the difference between the means of the upper and lower amplitudes. From these values, we calculated median, IQR, mean, 95% confidence intervals, and 95% prediction limits.

#### Sensitivity Analysis

A sensitivity analysis excluding patients with antiepileptic drugs (AEDs) was performed for the upper and lower amplitudes and bandwidths in order to rule out effects of AEDs on amplitude height.

#### Verification of Measurements and Interrater Reliability

Systematic measurement errors were ruled out by creating Bland-Altman plots for the upper and lower amplitudes of each channel (22). To assess interrater reliability, we calculated intraclass correlation coefficients for the upper and lower amplitudes using a two-way mixed model for individual ratings (23).

#### Software

SAS Enterprise Guide 8.4 (SAS Institute Inc., Cary, NC, USA) was used to perform statistical analyses and produce figures.

### Ethics Approval

The study was approved by the local ethics committee of the Medical Faculty of the University of Duisburg-Essen (20-9444-BO). Informed consent was not necessary according to local legislation, because data were anonymized.

## Results

Out of 98 performed cEEGs during the study period, 47 met the inclusion criteria. Seven cEEGs were excluded after cEEG review because abnormalities were identified. The duration of the remaining 40 recordings ranged between 14′48′′ and 23′30′′ (median 20′11′′). The median age at recording was 9.9 years (IQR 6.1–14.7 years) with 13 (33%) male patients.

All patients were admitted to the peripheral pediatric neurology ward electively in stable condition for the conduction of long-term cEEG. Indications for cEEG were suspected seizures in 22 (55%) patients, confirmed seizures or epilepsy in 16 (40%), and discontinuation of antiepileptic treatment in 2 (5%) Patients. Sixteen (40%) of patients received AEDs at the time of recording. Further information on patients, cEEG findings, and AEDs is presented in Table 1.

**Table 1:** Clinical information.

	*N* (%)^*^
Age (years) [median (IQR)]	9.9 (6.1–14.7)
Mean ± SD	9.9 ± 5.1
Sex male	13 (33%)
**Indications for EEG**	
Suspected seizures or epilepsy	20 (50.0%)
Treatment control	16 (40.0%)
Discontinuation of AED treatment	2 (5.0%)
Unknown	2 (5.0%)
**EEG findings**	
Unremarkable	26 (65.0%)
Solitary focal ETPs	6 (15.0%)
Solitary generalized IED	7 (17.5%)
Solitary beta waves	1 (2.5%)
Patients with AED treatment	16 (40.0%)
Monotherapy	10 (25.0%)
Combination therapy (2 drugs)	5 (12.5%)
Combination therapy (3 drugs)	1 (2.5%)

* *Unless indicated otherwise.*

*IQR, interquartile range; SD, standard deviation; AED, antiepileptic drugs; IED, interictal epileptiform discharges*.

### aEEG Patterns and Sleep States

All tracings displayed continuous background patterns according to Hellström-Westas (13). We observed simultaneous changes of the upper and lower amplitudes and bandwidth during the recording in all patients and in all analyzed channels (Figure 2). The values measured for the C3–C4 channel will be named in the following, as this is a typical position for single-channel aEEG. Detailed values of all analyzed channels and during all measured sections are given in Table 2.

**Table 2:** Observed amplitude values and prediction limits for each channel.

		**Wakefulness**			**Sleep**					
					**High amplitude-section**			**Low amplitude-section**		
**Channel**		**Lower (μV)**	**Upper (μV)**	**Bandwidth (μV)**	**Lower (μV)**	**Upper (μV)**	**Bandwidth (μV)**	**Lower (μV)**	**Upper (μV)**	**Bandwidth (μV)**
C3–C4	Median (IQR)	13 (10–19)	35 (27–49)	29 (21–34)	35 (25–44)	99 (71–125)	63 (44–81)	16 (11–20)	39 (30–51)	23 (19–31)
	95% PL	7–31	19–75	11–45	8–65	22–184	13–120	6–27	13–69	7–43
P3–P4	Median (IQR)	20 (16–25)	49 (40–62)	29 (23–38)	34 (24–47)	95 (68–124)	59 (44–76)	17 (13–20)	42 (35–51)	25 (22–31)
	95% PL	9–31	23−79	13–48	9–63	26–168	16–106	8–26	21–67	12–42
C3–P3	Median (IQR)	16 (10–20)	36 (26–46)	23 (16–27)	28 (18–36)	82 (51–102)	52 (33–62)	12 (9–16)	34 (25–44)	21 (15–28)
	95% PL	4–26	11–62	11–45	5–51	15–141	9–91	5–20	11–58	5–38
C4–P4	Median (IQR)	16 (11–21)	39 (29–53)	25 (17–30)	28 (21–37)	80 (56–104)	52 (35–66)	13 (10–16)	36 (28–45)	23 (17–28)
	95% PL	5–28	13–67	8–40	4–55	11–154	7–100	5–21	13–60	7–40
Fp1–Fp2	Median (IQR)	18 (13–22)	81 (57–93)	59 (44–74)	28 (21–34)	85 (66–102)	56 (45–68)	10 (8–13)	28 (22–34)	17 (13–20)
	95% PL	9–32	36–160	25–136	14–54	43–156	28–105	5–22	12–65	7–44

*PL, prediction limits*.

**Figure 3:**
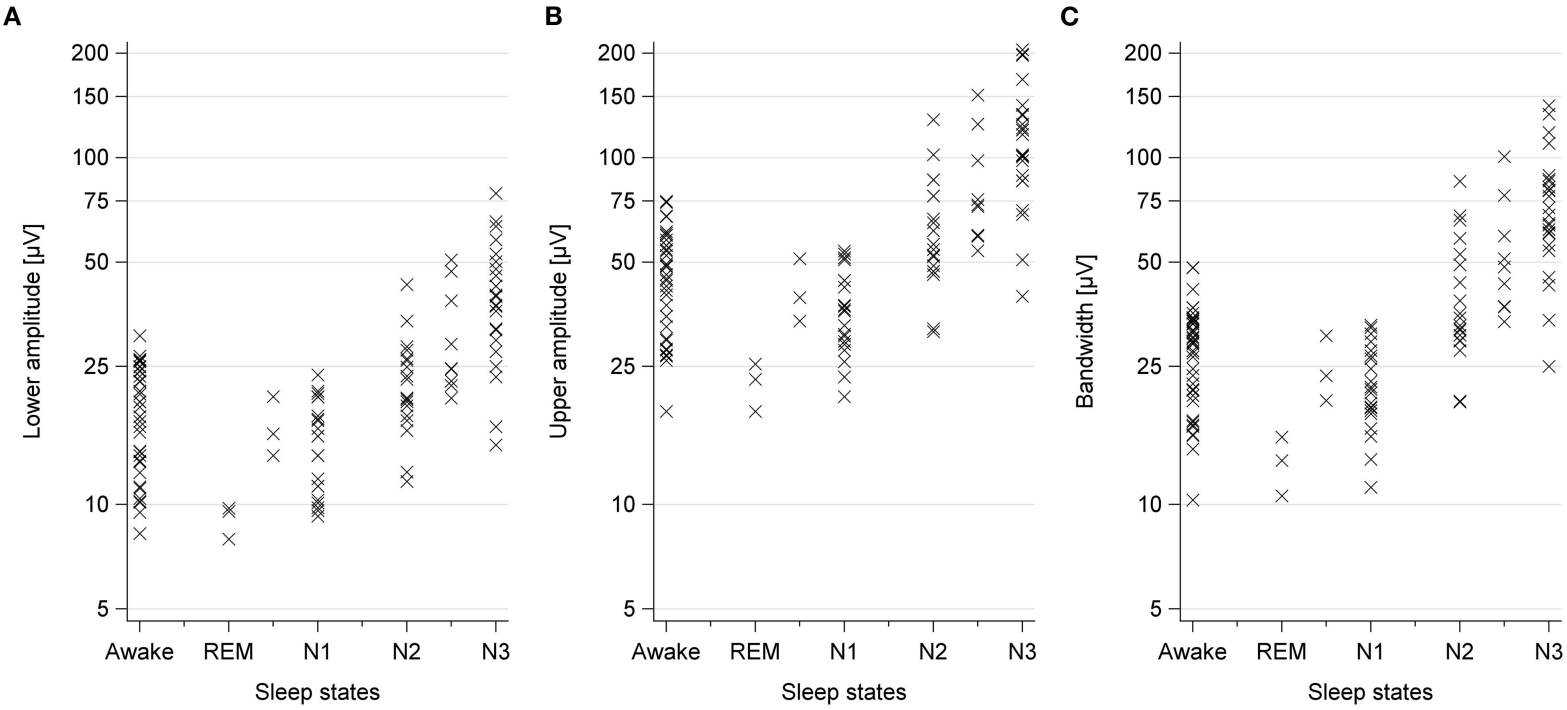
Observed amplitudes and bandwidths of the C3–C4 channel during wakefulness and different sleep states. Each symbol represents one measurement. **(A)** Upper amplitude. **(B)** Lower amplitude. **(C)** Bandwidth.

During wakefulness, the aEEG band remained constant with voltages of the upper border at 35 μV (IQR: 27–49 μV) and the lower border at 13 μV (10–19 μV). The bandwidth was 29 μV (21–34 μV). During sleep, the highest amplitudes [upper: 99 μV (71–125 μV), lower: 35 μV (25–44 μV), and bandwidths: (63 μV (44–81 μV)] occurred in the middle of the night, interrupted by short amplitude dips (Figure 2). Toward the morning, the low amplitude-sections became longer [upper amplitude: 39 μV (30–51 μV), lower amplitude: 16 μV (11–20 μV), bandwidth: 23 μV (19–31 μV)] (Figures 2, 3).

**Figure 4:**
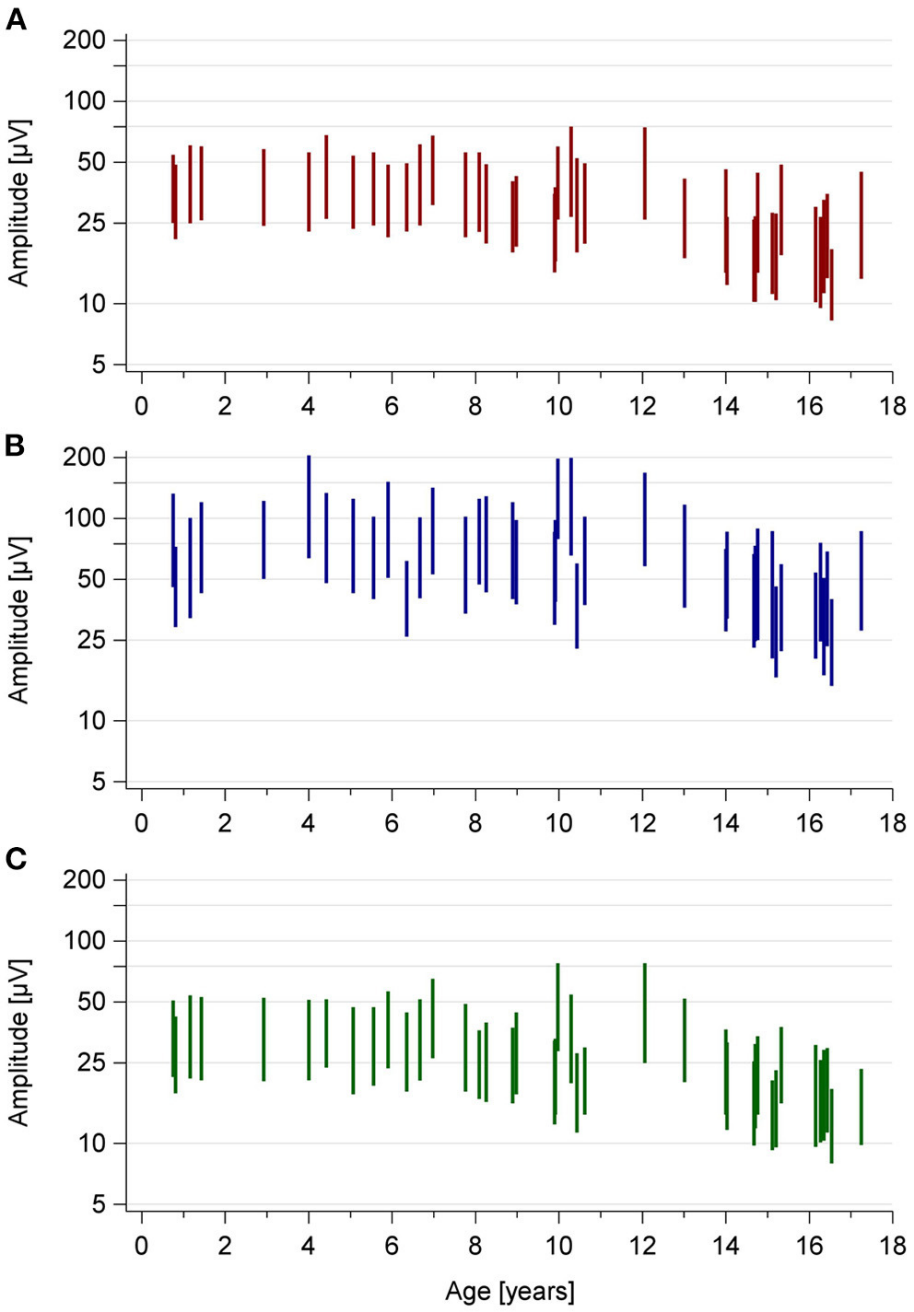
Amplitudes and bandwidths of each individual tracing by age. Each vertical line depicts the median values for amplitudes and bandwidth of one recording. The values are plotted using the same technique as aEEG devices: A line connects the value of the upper amplitude with the lower amplitude value, thereby representing the bandwidth. Instead of the time of the day, the x-axis represents the patient's age at the time of recording. This plot shows how that with increasing age aEEG values tend to be lower in older patients. **(A)** Wakefulness. **(B)** High amplitude-sleep section. **(C)** During the low amplitude-sleep section.

cEEG review revealed that high amplitude-sections were associated with sleep states N2 and N3, low amplitude-sections with wakefulness and sleep states REM, N1, and N2 (Table 3). One patient was awake during the low amplitude-episode at night. Amplitudes were slightly higher during wakefulness than during light sleep (Figure 3 and Table 2). With increasing age, amplitudes and bandwidths tended to be lower (Figure 4). No further age-specific analyses were conducted due to the small case number.

**Table 3:** Frequency of sleep states during high and low amplitude-sleep sections.

**Sleep state** **(according to AASM)**	**High** **amplitude-section**	**Low** **amplitude-section**
Awake	0	1 (2.4%)
REM	0	3 (7.3%)
REM or N1^*^	0	3 (7.3%)
N1	1 (2.4%)	22 (53.7%)
N2	7 (17.1%)	12 (29.3%)
Transition from N2 to N3	9 (22%)	0
N3	24 (58.5%)	0

*AASM, American Academy of Sleep Medicine; REM, rapid eye movement sleep.*

* *Not clearly distinguishable because due to unavailability of electro-oculogram*.

The distribution of sleep states during low amplitude-sleep episodes did not differ between patients with and without AEDs. During high-amplitude sleep episodes, patients without AEDs displayed sleep state N2 in 2 (8.3%), N2–N3 in 5 (20.8%), and N3 in 17 (70.8%) of cases. Patients receiving AEDs displayed N2 in 5 (31.3%), N2–N3 in 4 (25%), and N3 in 7 (43.8%) of cases. These numbers reflect the frequency of each sleep state during the assessed intervals, not the percentage of duration.

### Sensitivity Analysis

The sensitivity analysis showed no significant differences in upper and lower amplitude values and bandwidths between the whole study population and patients without AEDs (Supplementary Table 1).

### Interrater Reliability

Bland-Altmann plots showed no systematic differences in measurements between the two observers. The mean measurement difference (±SD) was 0.3 (±2.2) μV for the lower amplitude and 0.9 (±6.3) μV for the upper amplitude.

The intraclass correlation coefficient was 0.83 for the upper amplitude and 0.87 for the lower amplitude.

## Discussion

This study provides evidence of varying aEEG amplitudes between wakefulness and sleep states in non-critically ill children. We observed the highest amplitudes during deep sleep. Amplitudes during light sleep were slightly lower than during wakefulness. With increasing age, amplitudes tended to be lower, whereas we found no evidence that AEDs changed aEEG amplitude in the studied patients.

The changes in amplitudes we observed between wakefulness and sleep states can be explained by the raw EEG characteristics that define each sleep state: deep sleep (state N3) is characterized by high amplitudes and low frequencies (21). During wakefulness and sleep states N1 and REM, the EEG curve shows low voltages and high frequencies. Sleep state N2 is defined by high-amplitude K-complexes and sleep spindles (21). The voltage of the background activity can vary. This explains why sleep state 2 was detected during high and low amplitude-episodes.

Episodes with high amplitudes were interrupted by amplitude dips. In critically ill comatose or sedated children, we have previously observed high amplitudes without the ability to interpret this finding (1). Some of these children displayed intermittent amplitude dips, and some did not. Considering the results from this study, we assume that these dips can be interpreted as short transitions from deep to lighter sleep. Assessing aEEGs for amplitude dips during high amplitude-phases may help to identify patients with physiological sleep patterns in the PICU, while it must not be forgotten that critically ill children frequently display disrupted sleep wake-cycles. Whether the lack of dips is associated with critical illness itself, administration of sedation, specific clinical conditions, or even outcomes in pediatric critical care patients, demands further investigation.

A considerable proportion of patients in our study (40%) received AEDs during the recording. The sensitivity analysis showed no differences in amplitude values and bandwidths between the entire cohort and patients without AEDs. This information is relevant for pediatric intensive care givers, because more than half of critically ill children undergoing continuous EEG monitoring in the PICU receive AEDs (3). Among those who later display seizures, 95% are on AEDs at the time of EEG acquisition (3). We found no evidence in literature that sleep patterns are affected by AEDs, but children with epilepsy display sleep abnormalities more frequently than healthy children (24, 25). Therefore, children admitted to the PICU due to seizure disorders may be a subgroup requiring special attention upon aEEG interpretation.

Our study has several limitations. The low case number, inclusion of patients with epilepsy, and wide age range prohibits to use the measured values as reference values for pediatric aEEG. Subgroup analyses to determine age-specific values could not be performed due to the small sample size. Even though aEEG amplitudes did not differ between the whole cohort and patients without AEDs, the aEEG assessment may have missed minor differences in cEEG activity between patients with and without AEDs. Patients receiving AEDs displayed sleep state N2 during high amplitude episodes more frequently compared to children without AEDs. In children with epilepsy, loss of physiological sleep patterns has been described, with higher proportions of N2 sleep (25). As this study excluded tracings with serious pathologies and was not designed to assess the sleep states during the entire tracing, the distribution of sleep states we observed in patients with AEDs is likely not representative for children with epilepsy. Like in children with epilepsy, sleep patterns are altered in critically ill children as well. For this reason, the results cannot be generalized for the pediatric intensive care population but require cautious interpretation considering administered sedatives and other circumstances that affect electrocortical activity.

Our study provides evidence that aEEG amplitudes vary between wakefulness and different sleep states in stable children. This finding has important implications for clinical practice and further research on pediatric aEEG, as it shows that analysis of background patterns must take into account the state of wakefulness or sleep. Even though age-specific analyses were not calculated due to the small sample size, the decline in amplitudes in older children suggests that interpretation should also take into account the patients' age. Until reliable age-specific normal values have been established, the prediction limits from this study provide intensive care givers with approximate guidance what amplitude ranges to expect in awake and sleeping children: High amplitudes during deep sleep and lower amplitudes during wakefulness and light sleep.

## Data Availability Statement

The raw data supporting the conclusions of this article will be made available by the authors, without undue reservation.

## Ethics Statement

The studies involving human participants were reviewed and approved by Ethikkommission der Medizinischen Fakultät der Universität Duisburg-Essen. Written informed consent from the participants' legal guardian/next of kin was not required to participate in this study in accordance with the national legislation and the institutional requirements.

## Author Contributions

NB and AD: study design. VL, NB, and SG: conversion of aEEGs. VL and NB: analysis of aEEGs. AD: cEEG review for sleep state analysis. NB: statistics and drafting. UF-M, CD-S, HM, AD, SG, and VL: critical review and editing of the manuscript. All authors contributed to the article and approved the submitted version.

## Funding

The study received funding from the Medical Faculty of the University of Duisburg-Essen (Corona Care-Program) and from the Stiftung Universitätsmedizin Essen. NB received an internal research grant from the Medical Faculty of the University of Duisburg-Essen (IFORES) and a grant from the Stiftung Universitätsmedizin Essen.

## Conflict of Interest

The authors declare that the research was conducted in the absence of any commercial or financial relationships that could be construed as a potential conflict of interest.

## Publisher's Note

All claims expressed in this article are solely those of the authors and do not necessarily represent those of their affiliated organizations, or those of the publisher, the editors and the reviewers. Any product that may be evaluated in this article, or claim that may be made by its manufacturer, is not guaranteed or endorsed by the publisher.
