# Supplementary material for: Characterization of aEEG During Sleep and Wakefulness in Healthy Children
Source: Front Pediatr. 2022 Jan 21;9:773188. doi: 10.3389/fped.2021.773188 (PMC8814596; doi:10.3389/fped.2021.773188)
Supplement: Supplementary file 1 [file Table_1.docx]

Supplementary Table: Sensitivity analysis: means and 95% confidence intervals for all patients vs. patients without antiepileptic drugs

|  |  | Wakefulness | |  | |  | Sleep | |  |  | |  | |  | |  | |
| --- | --- | --- | --- | --- | --- | --- | --- | --- | --- | --- | --- | --- | --- | --- | --- | --- | --- |
|  |  |  |  | |  | | High amplitude-section | | | |  | | Low amplitude-section | | | |  |
| Channel |  | Lower (µV) | Upper (µV) | | Bandwidth (µV) | | Lower (µV) | Upper (µV) | | | Bandwidth (µV) | | Lower (µV) | | Upper (µV) | | Bandwidth (µV) |
| C3 – C4 | All  No AEDs | 19 (17 – 21)  20 (17 – 22) | 47 (42 – 51)  47 (41 – 53) | | 28 (25 – 31)  28 (24 – 31) | | 36 (32 – 41)  38 (32 – 43) | 103 (90 – 116)  104 (99 – 119) | | | 67 (58 – 76)  66 (57 – 76) | | 16 (15 – 18)  17 (15 – 19) | | 41 (37 – 46)  43 (37 – 49) | | 25 (22- 28)  26 (22 – 29) |
| P3 – P4 | All  No AEDs | 20 (18 – 22)  21 (18 – 23) | 51 (46 – 55)  52 (45 – 58) | | 31 (28 – 33)  31 (27 – 35) | | 36 (31 – 40)  38 (32 – 43) | 97 (85 – 108)  100 (86 – 114) | | | 61 (54 – 69)  63 (54 – 71) | | 17 (15 – 18)  17 (15 – 19) | | 44 (40 – 48)  44 (40 – 49) | | 27 (25 – 30)  27 (24 – 30) |
| C3 – P3 | All  No AEDs | 15 (13 – 17)  16 (14 – 18) | 37 (33 – 41)  38 (32 – 43) | | 22 (19 – 24)  22 (18 – 25) | | 28 (24 – 32)  29 (25 – 33) | 78 (68 – 88)  79 (69 – 89) | | | 50 (43 – 56)  50 (44 – 56) | | 12 (11 – 14)  13 (12 – 15) | | 34 (30 – 38)  36 (32 – 41) | | 22 (19 – 25)  23 (20 – 26) |
| C4 – P4 | All  No AEDs | 16 (14 – 18)  17 (15 – 19) | 40 (36 – 44)  41 (35 – 46) | | 24 (21 – 26)  24 (20 – 27) | | 29 (25 – 34)  30 (25 – 34) | 83 (71 – 94)  82 (70 – 94) | | | 53 (46 – 61)  53 (45 – 60) | | 13 (12 – 15)  13 (12 – 15) | | 37 (33 – 41)  37 (33 – 41) | | 23 (21 – 26)  24 (21 – 27) |
| Fp1 – Fp2 | All  No AEDs | 17 (15 – 19)  18 (16 – 20) | 76 (67 – 86)  81 (71 – 92) | | 58 (50 – 67)  62 (53 – 72) | | 27 (24 – 30)  27 (24 – 31) | 82 (74 – 91)  81 (72 – 92) | | | 54 (49 – 61)  53 (47 – 61) | | 11 (10 – 12)  11 (10 – 13) | | 29 (25 – 33)  30 (25 – 36) | | 18 (15 – 21)  19 (15 – 23) |

Data presented are mean and 95% confidence intervals
